# From Needs to Assets: Data-Driven Lessons from the Kenton County Age Well Initiative

**DOI:** 10.1093/geroni/igaf122.3594

**Published:** 2025-12-31

**Authors:** Ting Hong, Laura Allen, Michael Marcus, Dana Bradley

**Affiliations:** University of Maryland, Baltimore County, Baltimore, Maryland, United States; University of Maryland Baltimore County, Covington, Kentucky, United States; University of Maryland Baltimore County, Raleigh, North Carolina, United States; University of Maryland Baltimore County, The Erickson School of Aging Studies, Baltimore, Maryland, United States

## Abstract

This poster presents a case study of the Kenton County Age Well Initiative, a one-year, asset-based community development assessment project focused on leveraging assets to address the challenges of aging in Kenton County, KY. A first suburb of Cincinnati, Ohio, Kenton County is a geographically diverse community with a strong desire to support its older population, which is expected to grow from 16.5% of the population to 19% by 2030. The project began by forming an action committee of 25 local stakeholders to facilitate the team’s immersive understanding of individual, institutional, and association-based resources and county-specific challenges related to navigating aging services. Data were collected through community conversations, qualitative surveys, focus groups, and stakeholder interviews across the county with a total of 377 community members and 44 professionals. Four domains were identified as essential for supporting aging in Kenton County: housing, transportation, long-term care, and community vibrancy. Local demographic, organizational, and sector-specific data were then pooled and analyzed in each domain to identify assets and goals to address challenges. The project timeline is presented, highlighting key community engagement events from the project start to finish, including four county-wide workshops for professionals, older residents, and their caregivers. This poster details the lessons learned from the case study, including translating complex data sets to have high impact in community meetings, focusing on collaborative strategies for identifying key data points rather than relying solely on research team expertise, and reframing aging from deficit-based to asset-based in local planning efforts.

